# Insight into Biomass Upgrade: A Review on Hydrogenation of 5-Hydroxymethylfurfural (HMF) to 2,5-Dimethylfuran (DMF)

**DOI:** 10.3390/molecules26226848

**Published:** 2021-11-13

**Authors:** Nor Azam Endot, Ramli Junid, Mohamad Shazwan Shah Jamil

**Affiliations:** 1 Department of Chemistry, Faculty of Science, Universiti Putra Malaysia, Serdang 43400, Malaysia; 2 College of Engineering, Universiti Malaysia Pahang, Gambang, Kuantan 26300, Malaysia; ramli@ump.edu.my; 3 Department of Chemistry, Faculty of Science, Universiti Teknologi Malaysia, Johor Bharu 81310, Malaysia; shazwan.shah@utm.my

**Keywords:** hydroxymethylfurfural, dimethylfuran, hydrodeoxygenation, biomass, catalysis

## Abstract

Recent developments in the transformation of biobased 5-hydroxymethylfurfural (HMF) into a potential liquid fuel, 2,5-dimethylfuran (DMF), are summarised. This review focuses briefly on the history of HMF conversion to DMF in terms of the feedstock used and emphasises the ideal requirements in terms of the catalytic properties needed in HMF transformation into DMF. The recent state of the art and works on HMF transformation into DMF are discussed in comparison to noble metals and non-noble metals as well as bimetallic catalysts. The effect of the support used and the reaction conditions are also discussed. The recommendations for future work and challenges faced are specified.

## 1. Introduction

The growth in the demand for fossil feedstocks as the main source of chemicals and energy, together with their fast depletion, has increased the need for the development of a new and sustainable source of energy and platform chemicals. At the moment, fossils fuels contribute to nearly 92% of the commodity chemicals produced and 82% of energy sources. This is expected to change, and a large part of them can be generated from biomass in the near future [1]. Biomass is the fit candidate for alternative feedstock, given that it is abundant and the only widely available carbon source apart from oil and coal. In addition, the use of biofuels derived from biomass contributes to the mitigation of greenhouse gas emission, providing a clean and therefore sustainable energy source [2]. Biomass consists of carbohydrates, lignin, fatty acids, lipids, protein and others [3]. Depending on the source of the lignocellulose, the structure of hemicellulose varies and may consist of pentose, hexose and uronic acids. Lignin comprises 15–30% of lignocellulose. Like hemicellulose, lignin is a three-dimensional amorphous polymer consisting of methoxylated and phenylpropane structures [4]. In the plant cell wall, lignin fills the spaces between cellulose and hemicellulose and it acts like a resin that holds the lignocellulose matrix together. It is widely accepted that lignin is made up of the polymerisation of three monolignols, which are three different units of phenylpropane, namely *p*-coumaryl alcohol, coniferyl alcohol and sinapyl alcohol, that make it rich in aromatic functionality [5].

Owing to the different properties of each lignocellulose component, each one requires a different treatment or method of depolymerisation into its monomeric units, which, in turn, can be converted via fermentation or chemical routes to platform chemicals that can be used to produce fine chemicals, bio-derived monomers or fuels [6]. Examples of such platform chemicals are d-glucose (from the depolymerisation of cellulose); 5-hydroxymethylfurfural (HMF; from the dehydration of d-fructose), from which 2,5-dimethylfuran (DMF), 2,5-furandicarboxylic acid (FDCA) and levulinic acid (LA) are originated; and furfural (from the dehydration of C5 sugars) [7].

The catalytic conversion of biomass and derivatives to chemicals has been the theme of intense research efforts during the past decade, with a 20% annual increase in the number of publications on this topic [8]. Many publications focus on the large variety of biomass feedstocks and the reaction type. In 2004, the US Department of Energy (DOE) identified 12 building block chemicals that can be subsequently produced from sugars via biological or chemical conversions [9]. The 12 building blocks then can be further converted to several high-value biobased chemicals or materials.

Depending on the target products, hemicellulose and cellulose fractions are processed either together or separately. The simultaneous processing, such as gasification or pyrolysis, offers the benefits of simple separation, while the fractionation of hemicellulose and cellulose allows the processing of each fraction to be tailored to the advantage of the different physical and chemical properties of each fraction. Biomass consists of molecules typically highly functionalised, and this allows for many different chemicals to be produced; however, this also results in difficulties in controlling selectivity during the upgrade, as many parallel and consecutive reactions can appear, and, particularly, the degradation of the desired product or intermediate in the reaction medium [10]. Therefore, one of the most effective ways to process lignocellulosic biomass is through sequential steps that allow oxygen to be partially removed in the first step to reducing the reactivity of the feedstock. The second step is where the remaining functionality is modified to enable the upgrade to more valuable chemicals or fuels [11].

**Figure 1:**
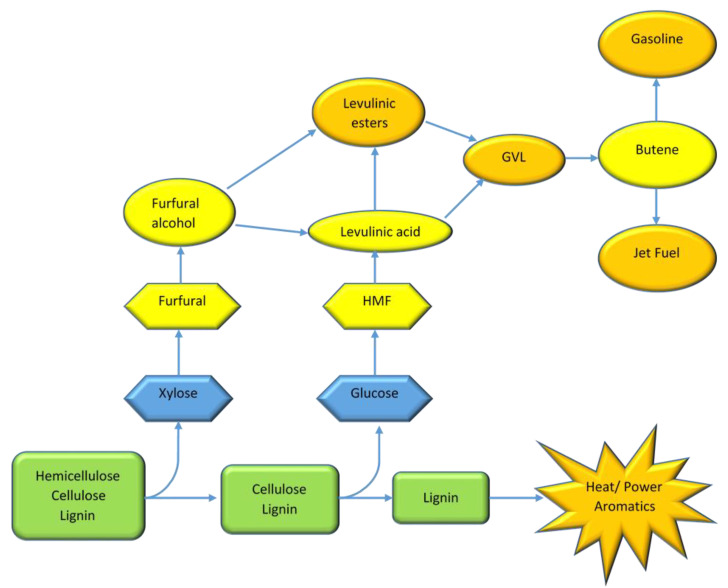
Roadmap for conversion of lignocellulosic biomass (green) to fuels (orange) and chemicals (yellow) passing through the intermediate formation of furfural and levulinic acid from C5 and C6 sugars (blue). Adapted from [12].

As illustrated in Figure 1, biomass can be upgraded to a variety of platform chemicals as well as fuels from C5 and C6 sugars. Chemical methods are usually used to process hemicellulose and cellulose after the fractionation, considering the different reactivities of C5 and C6 sugars—for example, the conversion of C5 sugars such as xylose to furfural or the conversion of the C6 sugar glucose to HMF, levulinic acid and formic acid. Among the chemicals obtained from xylose, the production of furfural has received major attention due to its potential to be converted into high-value-added chemicals, such as furfuryl alcohol, tetrahydrofuran or tetrahydrofurfuryl alcohol. Furfural is also used in oil refining, pharmaceutical, plastic and agrochemical industries [11]. In addition, furfural can be upgraded to platform chemicals and fuel precursors, such as levulinic acid and levulinic esters, through the intermediate furfuryl alcohol. Like furfural, HMF is a promising platform chemical derived from sugars but from the C6 fraction. This will be discussed in detail in Section 2.

**Figure 2:**
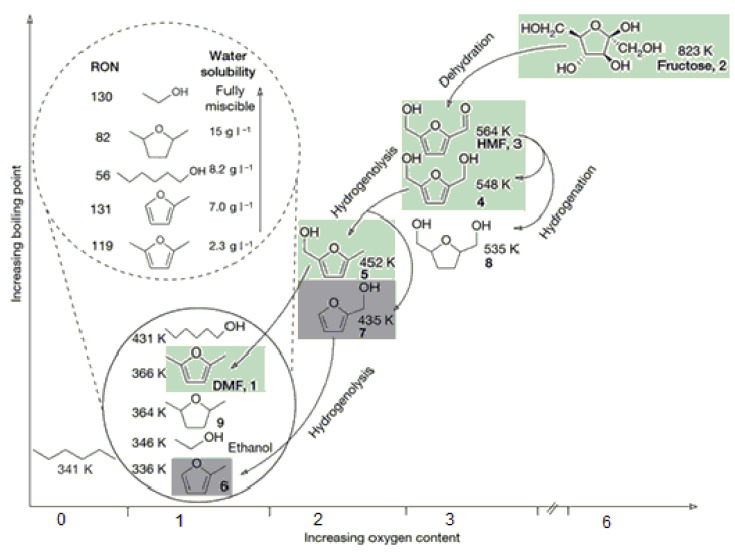
Normal boiling points of representative C6 hydrocarbons formed by removal of oxygen atoms from hexoses compared to the normal boiling point of ethanol. Adapted from [13].

The main drawback of biomass as a feedstock and, particularly, carbohydrates is the high content of oxygen within its molecular structures. Removing oxygen increases the energy density if the product is for fuel use. Figure 2 shows the selective removal of oxygen atoms from hexose (fructose) to produce DMF. It not only reduces the boiling point but also reaches the lowest water solubility and research octane number (RON) of mono-oxygenated C6 compounds, which are suitable for liquid fuels [13]. There are three main options for lowering the oxygen content in carbohydrates. The first option is the removal of small and highly oxidised carbon molecules, such as CO_2_, formaldehyde and formic acid. Fermentative conversion of carbohydrates to ethanol, butanol and CO_2_ is one of the examples. The second option is through hydrogenolysis, which is the removal of oxygen from the molecule by forming water. The third option is the removal of water by the dehydration of carbohydrates into a variety of interesting compounds, especially furans and levulinic acid [13].

This review focuses briefly on the history of HMF conversion to DMF in terms of the feedstock used and emphasises the ideal requirement in terms of the catalytic properties needed in HMF transformation into DMF. Furthermore, this review highlights the consolidation of catalysts used and the possible reactions that can take place in HMF hydrogenation into DMF from many studies into one paper. Subsequently, the recent state of the art and works on HMF transformation into DMF are discussed in comparison to noble metals and non-noble metals as well as bimetallic catalysts. The parameters that can affect the DMF yield and product selectivity are also discussed. Finally, recent progress on DMF production and prospects are also provided.

## 2. Production of HMF

**Figure 3:**
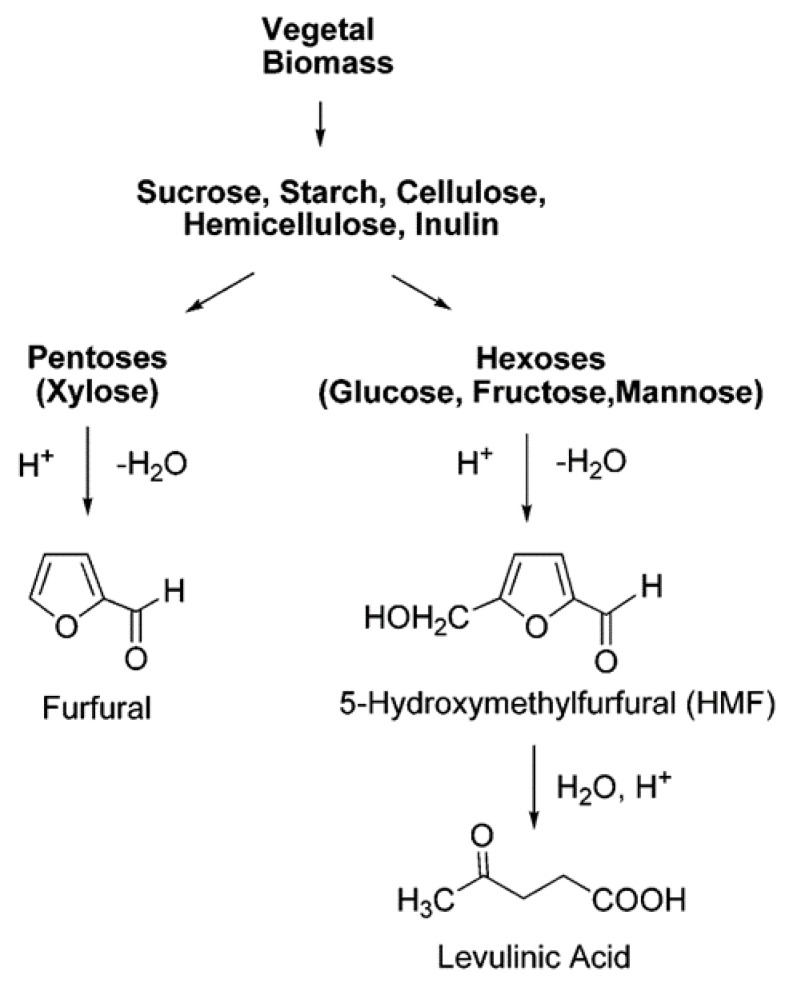
Products obtained from dehydration of monosaccharides.

The synthesis of HMF is based on the acid-catalysed triple dehydration of hexoses, mainly glucose and fructose. Although disaccharides or polysaccharides, such as sucrose, cellobiose, inulin or cellobiose, as well as converted industrial wastes, can be used as HMF sources, hydrolysis is necessary for depolymerisation (Figure 3) [14].

Besides HMF, the acid-catalysed dehydration reaction leads to at least traces of various other dehydration products, such as levulinic and formic acids and polymeric side products called humines or humic acids [15,16]. To prevent these side reactions and obtain a high yield of HMF, the proper design of catalysts selective to HMF is crucial. The use of a multifunctional catalyst based on transition metals with solid/base catalysts is expected to allow several reaction steps to be finished in one reactor and avoid the costly intermediate separation process [17]. In addition, the recycling of the catalyst and the efficient separation of the target product would also contribute to the efficient production of HMF.

**Figure 4:**
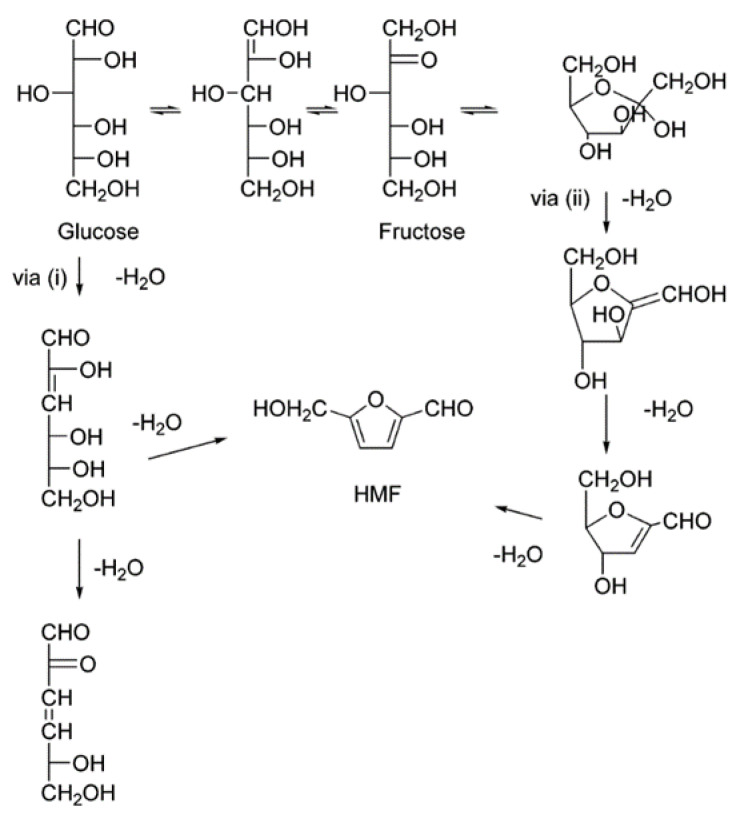
Pathways for the dehydration of hexoses to HMF.

The mechanism of HMF formation from fructose and sucrose has been described by Antal et al. [18], Van Dam [19] and Kuster. [20]. They concluded that the dehydration could occur via two possible pathways, as shown in Figure 4: (i) based on acyclic compounds and (ii) based on the transformation of ring systems. This also demonstrates that the chemistry of the formation of HMF is complex due to many reactions involved besides dehydration. For example, a series of side reactions, such as isomerisation, fragmentation and condensation, strongly influence the yield of the product. The production of HMF from the dehydration process is more efficient and selective from fructose than from glucose due to a low degree of glucose enolisation, owing to the stable ring structure of glucose compared to fructose. Enolisation, also known as tautomerisation, is a structural isomer in which a hydrogen atom is transferred from one carbon to another, as shown in Figure 4. Since enolisation is the rate-determining step of HMF formation, glucose will react slower than fructose. Moreover, glucose can condense to form oligosaccharides, which can react with HMF, resulting in cross-polymerised materials. Nonetheless, glucose is still used as a feedstock for HMF production due to its lower cost.

Reviews of HMF production and the kinetic studies of the dehydration reaction have been published [7,14,16,20,21]. The most convenient method of obtaining HMF is the acid-catalysed dehydration of fructose. Fructose can be obtained by acid-catalysed hydrolysis of sucrose and inulin or by selective isomerisation of glucose to fructose. Dehydration of hexoses to HMF has been carried out using a variety of catalysts, such as organic acid (oxalic, maleic acids), inorganic acids (H_2_SO_4_, HCL), organic and inorganic salts and solid acids, Lewis acids (ZnCl_2_, AlCl_3_) and others (ion-exchange resins, zeolites) [22].

## 3. DMF as an Alternative Fuel

2,5-Dimethylfuran (DMF), among other furan derivatives, such as 2-methylfuran (MF) and furan, is a promising biomass-derived renewable fuel candidate to reduce the consumption of fossil fuels and engine emissions. This is due to its combustion properties comparable to those of commercial gasoline and the sustainable productivities from lignocellulosic raw materials. The use of biofuels, such as methanol and ethanol, that have been blended with gasoline as transportation fuels in many countries could greatly reduce the dependence on fossil fuels [23]. However, methanol and ethanol have lower heating values that increase the transportation costs, and their high solubility in water poses a threat to water security. These fundamental disadvantages limit the practical use of bioethanol [24,25].

DMF has received significant attention due to its high energy density (30 MJ/L), high research octane number (RON = 119), low oxygen content (O/C = 0.17) and ideal boiling point (92–94 °C). In addition, DMF is nearly immiscible in water and thus easier to blend with gasoline than ethanol. Moreover, DMF has a low latent heat of vaporisation (0.30 kJ cm^−3^), which means lower energy consumption during purification through distillation compared to bioethanol [26]. A comparison of the fuel properties of DMF with those of ethanol and gasoline is shown in Table 1.

Recently, DMF-blended gasoline was tested on a single-cylinder gasoline direct injection gasoline engine against the benchmark of standard gasoline. The test results revealed that DMF has satisfactory combustion, ignition and emission characteristics comparable to commercial gasoline [28,29]. A detailed review of the fundamental combustion characteristic of DMF and furan derivatives was published by Xu et al. [28].

**Figure 5:**
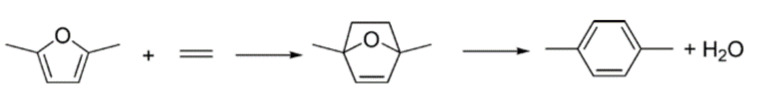
Production of ρ-xylene via Diels–Alder cycloaddition with ethylene and subsequent dehydration.

DMF has the potential for mass production from an abundant raw material, such as lignocellulose biomass [30]. Furthermore, it is possible to obtain high efficiency in the conversion of DMF from biomass, which allows DMF to be used as an alternative fuel candidate [16]. Recently, DMF has been recognised as a source of biobased ρ-xylene production via Diels–Alder cycloaddition with ethylene and subsequent dehydration (Figure 5) [31,32,33].

## 4. Production of DMF

The first process for the production of DMF was reported in 1980 by Saha et al. [27]. It involves two-step processes, where, firstly, HMF is reduced to 2-methylfurfural alcohol (MFA) using hydrazine (N_2_H_4_), followed by hydrodeoxygenation using Pd/C and cyclohexene as a hydrogen source to produce 27% DMF at 80 °C. Since then, this technology has not been explored until the emergence of DMF as a sustainable biofuel was established. Many researchers have now developed catalytic routes to produce DMF selectively, and this has recently been reviewed [27].

**Figure 6:**
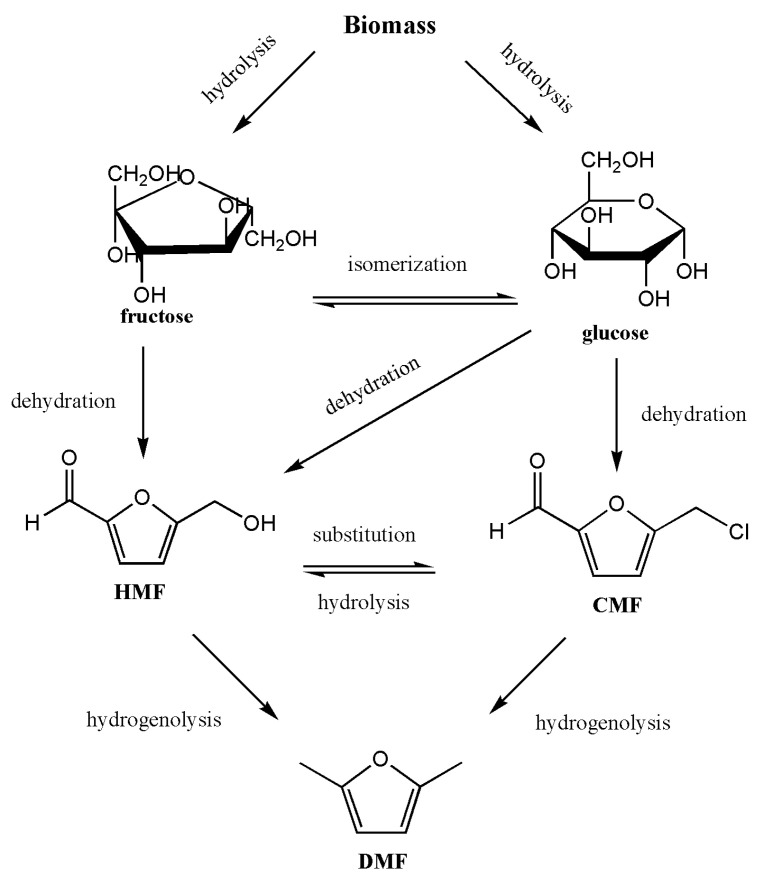
Pathway of DMF preparation from biomass. Adapted from [35].

The typical processes to acquire upgraded biofuels or chemical platforms require the use of homogeneous, heterogeneous or biological (enzymes, microorganisms and yeasts) catalysts [3]. The chemical process of transforming lignocellulose into DMF is a multi-step process. The first step typically involves the pre-treatment of lignocellulose to glucose, followed by the acid-catalysed dehydration of the glucose isomer, fructose, into HMF. The next step is the catalytic hydrodeoxygenation (HDO) of HMF into DMF [34]. Figure 6 illustrates the pathway of DMF preparation from biomass.

Recently, Dutta et al. demonstrated that DMF can be produced from 5-chloromethyl furfural (CMF) instead of HMF over Pd/C (2:1 N,N-dimethylformamide/acetic acid solvent mixture, room temperature, 3 atm H_2_, 1.25 h) [36]. CMF is produced under mild conditions and in a high yield from sugars, cellulose or directly from cellulosic biomass hydrochloric acid treatment in a biphasic system [37].

A thermodynamic study by Verevkin et al. revealed that hydrogenation of HMF to DMF is feasible through hydrogenation of HMF to 2,5-dihydroxymethylfurfural (DHMF) and subsequent hydrogenolysis of DHMF to DMF, given the equilibrium constant completely shifts to the desired reaction product even at 25 °C [38].

Recent advances have allowed the production of DMF from a different source of HMF by acid-catalysed hydrolysis of cellulose into sugar components, for instance, glucose and fructose. Dumesic et al. performed the synthesis of DMF with 71% yield by hydrogenation-hydrogenolysis of HMF from fructose using Cu-Ru/C catalyst [13]. Binder and Raines used untreated corn stover to synthesise DMF [30]. They used CrCl_3_-HCl catalyst to transform corn stover into HMF, followed by hydrogenation-hydrogenolysis of HMF into DMF with Cu-Ru/C catalyst under H_2_.

**Scheme 1:**
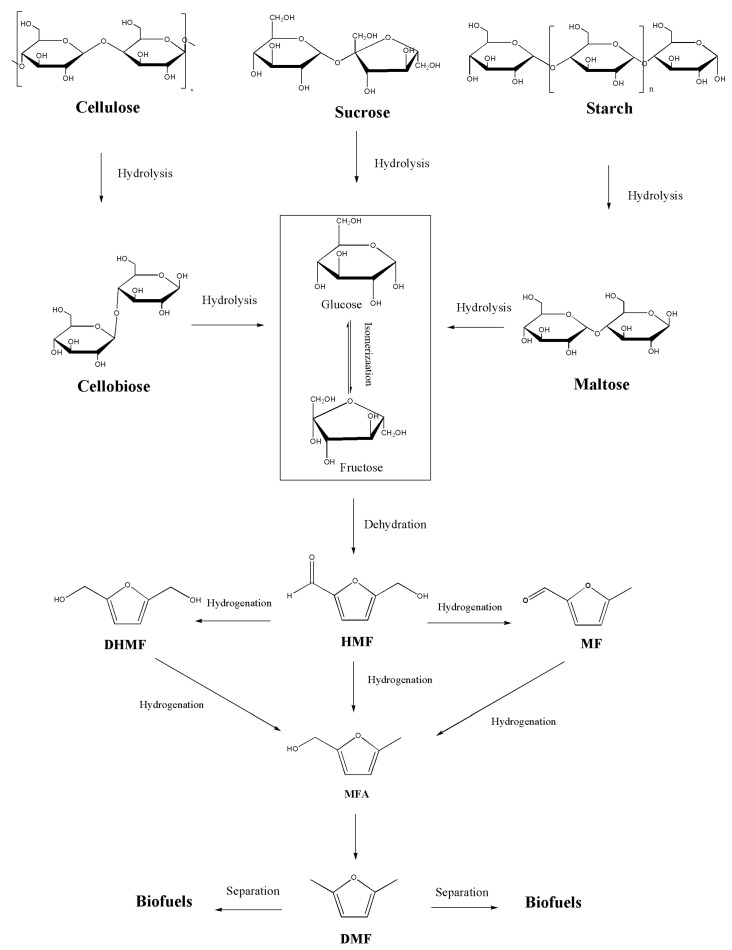
Integrated reaction pathway for the transformation of biomass-derived carbohydrates into DMF. Adapted from [40].

The production of DMF from fructose via one-pot synthesis was investigated by Sudipta et al., with a maximum yield of 32% DMF [39]. The reaction progressed via HMF synthesis using formic acid as a catalyst for fructose and N,N-dimethylacetamide (DMA) catalyst for glucose and untreated biomass in the first step. In the subsequent steps, HMF was converted to DMF by hydrogenation and hydrogenolysis reactions using Ru/C catalyst and formic acid as a H_2_ source. Scheme 1 illustrates the integrated reaction pathway for the transformation of biomass-derived carbohydrates into DMF.

## 5. Mechanism of HMF Hydrogenation to DMF

**Scheme 2:**
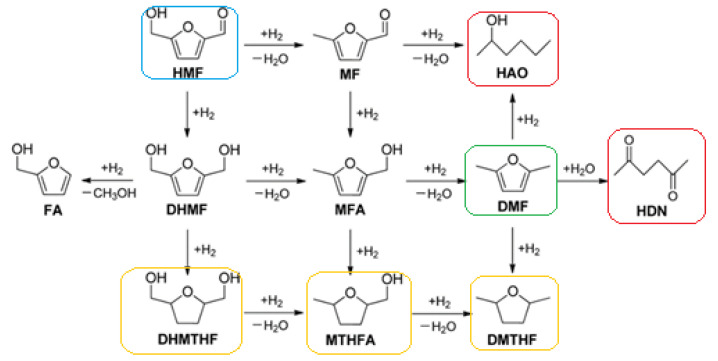
Possible reaction mechanism for the hydrogenation of HMF to DMF. Adapted from [40].

There are many types of catalytic reaction mechanisms proposed by researchers for the selective hydrogenation of HMF to DMF, but they always involve three consecutive steps, as summarised by Hu et al. [40] (Scheme 2). The first step involves the hydrogenation of the C2 aldehyde group and the C5 hydroxyl group in HMF into 2,5-dihydroxymethylfurfural (DHMF) and 5-methylfurfural (MF), respectively. The presence of the conjugated field on the furan ring stabilises the ring, and the electron-withdrawing effect of oxygen on the aldehyde group makes the C2 aldehyde group and the C5 hydroxyl group unstable. Conjugation allows a delocalisation of pi electrons across the adjacent p orbitals, which in general may lower the overall energy of the molecule and increase stability [41]. Furan is a five-membered ring with two alternating double bonds and oxygen in position 1. Oxygen has two lone pairs, one of which occupies a p orbital on that position, thereby maintaining the conjugation of that five-membered ring. Nishita et al. found that the conversion of HMF to DHMF is more prominent compared to HMF into MF using Ru-K-OMS-2 catalyst [42]. This is due to the selective adsorption of HMF through the carbonyl group, which results in the formation of DHMF. Then both DHMF and MF are subsequently hydrogenated into the same intermediate 5-methylfurfuryl alcohol (MFA). The last step is the hydrogenation of MFA to the targeted product 2,5 dimethylfuran (DMF).

Although from the thermodynamic point of view, the C=C bond is easily hydrogenated compared to C=O, the presence of the conjugated furan ring makes the hydrogenation of the C=O bond relatively easier than the C=C bond in the selective hydrogenation of HMF to DMF [41]. Thus it is possible to obtain high selectivity towards DMF. However, many other possible by-products and intermediates can be generated due to the high reactivity of HMF, such as furfuryl alcohol (FA), 2-5-dihydroxymethyltetrahydrofuran (DHMTHF), 5-methyltetrahydrofurfuryl alcohol (MTHFA), 2,5-dimethyltetrahydrofuran (DMTHF), 2,5-hexanedione (HDN) and 2-hexanol (HAO). Under appropriate conditions, such as reaction temperature, H_2_ pressure, time of reaction and catalyst, the formation of this by-product is less favourable. 

A recent study by Lei et al. showed that various parameters play important roles in the formation of high DMF yield [40]. Among the metals supported on carbon (Pd, Pt, Rh), Ru exhibits excellent reactivity, with 80.6% yield after 2 h. In has been found that 5 mol % Ru/C loading is the optimum loading for high DMF yield [43,44]. As for the reaction time, 2 h was found to give the highest DMF yield. When the reaction time was prolonged, the yield of DMF decreased, which demonstrated the formation of undesired by-products. Increasing the H_2_ pressure raised the solubility of H_2_ in a solvent, thus facilitating the hydrogenation of HMF to DMF. However, at low H_2_ pressure, the yield of DMF was low due to incomplete hydrogenation of HMF to DMF, which possibly results in the formation of intermediates such as DHMF and MF. Too high H_2_ pressure might promote further hydrogenation and the opening of DMF, resulting in by-products such as DMTHF and HAO.

A study by Mitra et al. found that product distribution is highly dependent on the reactant concentration and catalyst loading [44]. Increasing the concentration of HMF by 4-fold led to a decrease in ring hydrogenation products and improved the selectivity of DMF. Catalyst loading also influences the selectivity of hydrogenation/hydrogenolysis of the ring. Decreasing catalyst loading from 10 to 5 mol % decreased the tendency for ring hydrogenation before hydrogenolysis, as confirmed by the absence of DHMTHF in the reaction.

## 6. Catalytic Conversion of HMF to DMF

As one of the versatile compounds, HMF is the key to the production of DMF. The conversion of HMF to DMF has been widely studied, and the recent progress is summarised in Table 2. 

Among the works on HMF hydrogenation reported in the literature, it can be seen that most of the catalysts used consist of a noble metal, such as Pd, Ru or Pt, apart from transition metal catalysts based on Cu, Ni and Co supported on metal oxides and carbon. The addition of a secondary metal from a transitional metal to a noble metal has also been used by many researchers. Apart from the different types of metal, it was also observed that reaction conditions, namely the type of solvent, H_2_ donor, reaction temperature, reaction time and H_2_ pressure, play an important role in influencing the HMF conversion and DMF yield.

Dumesic et al. were able to produce a reasonable DMF yield using CuCrO_4_ and CuRu/C catalysts under a batch condition in 1-butanol at 220 °C and 6.8 bar H_2_ in 10 h (entry 2). It was observed that the addition of Ru to Cu supported on carbon increased the DMF yield from 61% to 71% under the same reaction condition. Although the Ru/Co_3_O_4_ (entry 13)-catalysed reaction achieved a high DMF yield (93%) at relatively low pressure and temperature, a high catalyst loading (40 wt% to HMF) and long reaction time (24 h) are needed. Pd/C in supercritical carbon dioxide leads to 100% yield of DMF in 2 h; however, the reaction requires a specific combination of water and supercritical CO_2_ [entry 12]. 

DMF production by catalytic transfer hydrogenation has also been investigated using formic acid (FA) [39,43], methanol [44] and isopropyl alcohol (IPA) [45,53]. Thananatthanachon et al. demonstrated the conversion of HMF to DMF using Pd/C/H_2_SO_4_ (2 mmol HMF in THF, 0.4 g of catalyst) with 95% yield of DMF in 15 h at 120 °C [47]. However, this requires the addition of H_2_SO_4_. Dutta et al. performed the same reaction using Ru/C; however, a lower DMF yield was obtained (30%) [36].

Jae et al. investigated the catalytic transfer hydrogenation (CTH) of HMF into DMF with 2-propanol as a hydrogen donor over Ru/C and RuO_2_ [68]. They found that the catalyst for the CTH of HMF into DMF is a bifunctional material consisting of both Ru and RuO_2_. It was suggested that the synergy of Ru and RuO_2_ is responsible for the selective production of DMF.

### 6.1. Hydrogenation with Noble Metal Catalysts

Typical processes to acquire upgraded biofuels or chemical platforms require the use of homogeneous, heterogeneous or biological (enzymes, microorganisms and yeasts) catalysts [3]. This process normally involves acid-catalysed reactions. As the conversion of HMF to DMF is a hydrogenation/hydrogenolysis process, catalysts used for this reaction are hydrogenation catalysts, commonly noble metals such as Pd [50], Ru [39,42,51], Pt [52] and others, which are generally supported on carbon in some form. This combination provides high reactivity and good dispersion on sustainable and tuneable supports with a high surface area and easy-to-modify porosity and surface properties. 

However, noble metals may be deactivated in water, which is commonly used in these hydrogenation reactions. Moreover, their price is high and unsustainable. Only in the case of low concentrations and high catalyst lifetime can these catalysts be considered as those of the future for biomass transformations. However, they possess high reactivity even at low hydrogen pressures, and it was shown that it is possible to obtain 100% HMF conversion and 98% DMF yield after 2 h at 180 °C and 10 bar H_2_ over Pt-Co in hollow carbon nanospheres [52]. In another study, Ru/Co_3_O_4_ catalyst exhibited 93.4% DMF yield at 130 °C and 7 bar H_2_ [51]. Further still, transfer hydrogenation with 2-propanol as a hydrogen donor was carried out by Jae and co-workers, who used Ru/C and achieved 100% HMF conversion and 81% DMF selectivity after 6 h at 190 °C under 20 bar N_2_ [45]. With the same catalyst, Ru/C, in the presence of THF as the solvent yielded 94.7% DMF at 200 °C under 20 bar H_2_ for 2 h [43]. 

### 6.2. Hydrogenation Reactions with a Transitional Metal Catalyst 

To date, most of the catalysts being used in the transformation of HMF into DMF involved precious metals such as Ru, Pt and Pd [27]. Thus an alternative catalytic system based on non-precious metals (Co, Ni, Cu and Fe) is crucial from the economic point of view since they are cheaper. 

Ni and Co are typically active metals for hydrogenation processes since both metals can hydrogenate C=C and C=O bonds [60,69]. Raney nickel catalyst, for example, has been used in a wide range of hydrogenation reactions, such as in the hydrogenation of nitro compounds, alkenes, carbonyl compounds, nitriles, alkynes and aromatic compounds [70]. Recently, Iriondo et al. demonstrated that Cu catalyst supported on ZrO_2_ has the best selectivity towards DMF among other compared metals such as Pt and Ru [71]. Ni supported on Co_3_O_4_ also shows good activity as an additive to Co_3_O_4_ in converting HMF to DMF, since both of the elements have a good ability to break C–O bonds [58]. A 76% yield of DMF was achieved under relatively mild reaction conditions (130 °C, 10 bar H_2_, 24 h). Kong et al. showed that Ni supported on Al_2_O_3_ is promising for HMF hydrogenation, with a high yield of DMF, DMTHF and DHMTHF [60]. The modulation of the surface metal–acid bifunctional site via calcination temperatures and control reaction conditions resulted in a high yield of DMF (91.5%), DMTHF (97.4%) and DHMTHF (96.2%).

**Figure 7:**
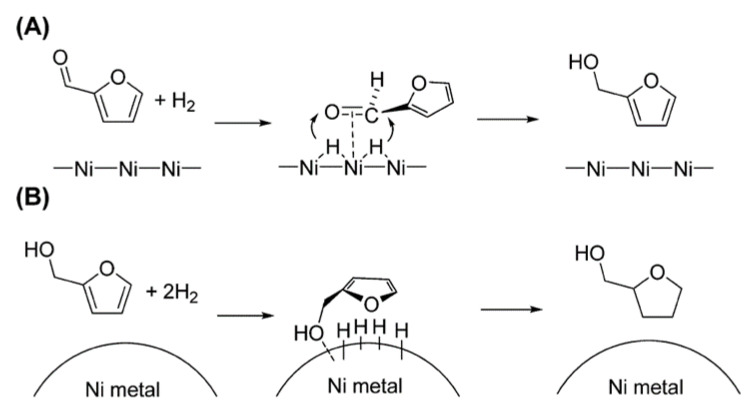
Proposed mechanisms for the hydrogenation of (**A**) furfural and (**B**) FOL over Ni/SiO_2_ [72].

Catalysts based on Ni and Co were also used in the chemoselective hydrogenation of furfural. Furfural hydrogenation is quite similar to HMF hydrogenation, apart from having less methyl and a hydroxyl group [71]. Ni/SiO_2_ was reported to have better reactivity than Rh/SiO_2_ for the hydrogenation of furfural and selectivity to furfuryl alcohol [72]. Figure 7 shows the schemes proposed by Nakagawa et al. for the hydrogenation mechanism of furfural and furfural alcohol over Ni/SiO_2_ catalyst. Based on the kinetic analysis, they reported that adsorption of furfural on Ni/SiO_2_ catalyst is much stronger than that of furfural alcohol (FOL) under hydrogenation conditions, suggesting that furfural is adsorbed at the C=O group [73].

The use of new and cheap metals for the hydrogenation of HMF to DMF should be explored more since this could be beneficial as an alternative for the precious metal. Apart from the transition metal being used as a monometallic catalyst for hydrogenation, the combination of these metals with other transition metals or noble metals is also getting more attention [27].

### 6.3. Hydrogenation Reactions with Bimetallic Catalysts 

Bimetallic catalysts have been receiving a lot of attention in selective oxidation [74], Fischer–Tropsch [75] and hydrogenation catalytic [76] reactions. This is due to positive synergistic effects emerging upon alloying that result in structures and properties that are distinct from those of the pure element. The chemical and physical properties may be tuned by varying the composition and atomic ordering as well as the size of the clusters. In fact, bimetallic metal nanoparticles may display not only magic sizes but also magic compositions at which the alloy presents special stability that may determine the chemical reactivity, especially catalytic activity [77]. For instance, in benzene hydrogenation, Yoon et al. [78] synthesised a bimetallic catalyst of Pd-Rh/CNT via a microemulsion method, and it was found that this combination exhibits the highest TOF compared to its monometallic analogues. Another example was shown by Zhu et al., who showed that Ru-Ni/C (0.024 wt% Ru, 1.00 wt% Ni) shows the highest TOF for hydrogenation of benzene compared to monometallic Ni/C and Ru/C [76]. The authors suggested that this is due to the efficient synergistic effect between Ru, Ni and NiO sites, stemming from the nanostructure of Ru on Ni/NiO nanoparticles. It is also speculated that the additional metals can improve the size and morphology of active particles as well as the catalysts’ selectivity [79].

In the hydrogenation of glucose to sorbitol, the addition of metalloids (elements that have properties of both metals and non-metals, such as boron, B) as the promoter to Ni demonstrated an improvement in activity. The authors found that the higher activity of this alloy catalyst is due to the combination of the structural features and the surface electronic state [80]. Extended X-ray absorption fine structure (EXAFS) analyses of the samples revealed that the amorphous catalyst has a lower Ni coordination number and a shorter Ni–Ni bond distance compared to a crystalline catalyst that has been calcined. This was considered to be responsible for the active catalyst, which is beneficial for hydrogenation reactions. Furthermore, the addition of B to Ni makes it electron-rich, and as a consequence, the glucose adsorption through the C=O group is weakened. This means that more H_2_ could be adsorbed on the Ni-B catalyst, and hence a higher hydrogenation activity is observed [80]. 

Besides improving the reactivity of the reaction, the bimetallic system also can help in reducing the dependence on noble metals by incorporating some non-noble metals, such as Ni and Co. Not only they are cheaper, but in certain molar ratios, they could result in better reactivity than the noble metals themselves [81].

As for the hydrogenation of bio-derived furan derivatives such as furfural and HMF, many Ni-, Co- or Cu-based bimetallic catalysts have been used [55]. Recently. Chen et al. demonstrated that carbon-coated Cu-Co bimetallic nanoparticles show excellent performance in selective hydrogenolysis of HMF to DMF, with 99.4% yield of DMF at 180 °C and 50 bar H_2_ in 8 h [61]. X-ray photoelectron spectroscopy (XPS) analysis revealed the coexistence of cobalt oxide species that are responsible for the synergistic effect between cobalt species and copper, while Luo et al. reported that (10 wt%) Pt-Ni alloyed nano-crystals supported on carbon with a ratio of 3:1 exhibit a high yield of DMF compared to other compositions and its monometallic catalysts [63]. Ni-Fe/CNT (10 wt%) also displayed high selectivity towards DMF and DHMF, depending on the temperature. This was attributed to the formation of Ni-Fe alloys species that is beneficial to the C–O bond cleavage [64]. The bimetallic non-noble metals were also demonstrated by Giovanni et al. A high yield of the mixture of both DMF and DMTHF was obtained when Cu-Zn nanoalloy was used in HMF hydrogenation [66]. The authors proposed that the synergistic effect between active CuO sites with Lewis acidic ZnO sites is responsible for the high reactivity based on a previous study [82].

**Figure 8:**
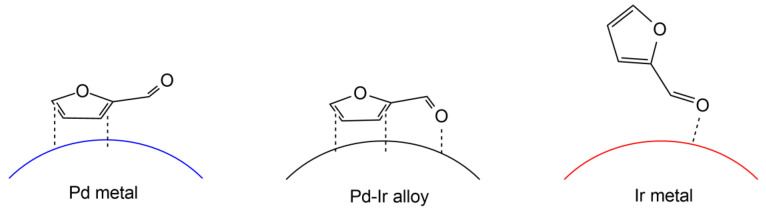
The proposed adsorption structure of furfural on the catalyst surface. Adapted from [83].

Figure 8 illustrates the adsorption structure of furfural on the catalyst surface, as proposed by Nakagawa et al. [83]. It was believed that the addition of Ir may promote the adsorption of the C=O site on furfural and weaken the adsorption on the furan ring in the case of Pd-Ir/SiO_2_ catalyst. This was demonstrated by comparing the selectivity of furfural and furfuryl alcohol on Pd-Ir alloy to monometallic Pd and Ir. The high reactivity of furan ring hydrogenation was observed on monometallic Pd. In the presence of furfural, the hydrogenation of furfuryl alcohol over Ir was suppressed, suggesting the strong adsorption of C=O on the surface of Ir.

**Figure 9:**
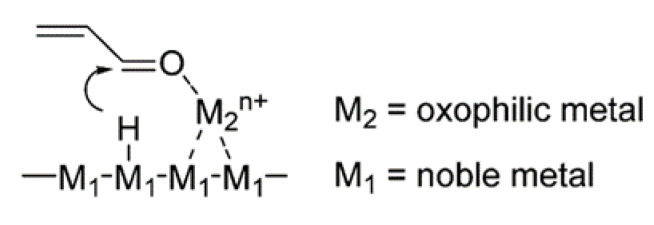
The proposed adsorption structure of furfural on the catalyst surface.

The property of the adsorption of the substrate on the catalyst can be changed by the addition of a secondary metal, particularly an oxophilic metal (electropositive element) such as Sn. Vetere et al. [72] studied the chemoselective hydrogenation of furaldehyde using monometallic Pt, Ni or Rh and a bimetallic catalyst with Sn supported on SiO_2_. They found that the addition of Sn shows significant conversion for Pt and an increase in selectivity towards furfuryl alcohol with Ni. Figure 9 shows the promotion effect of an oxophilic metal on substrate adsorption at the C=O bond [84,85,86]. The presence of an oxophilic metal or cation on the noble metal surface induces the hydrogen species formed on the noble metal to be transferred to the C=O bond to achieve selective hydrogenation.

After the recent increased interest in biomass-related transformations, the challenges for the catalysis chemist reside in the complex structure and functionalities of biomass. The challenge in designing catalysts to facilitate the new transformations is to prepare catalysts that can selectively remove functional groups and break specific chemical bonds in the biomass-derived feedstock. Thus, bimetallic catalyst systems seem to be promising due to the synergistic effects between two combination metals.

**Figure 10:**
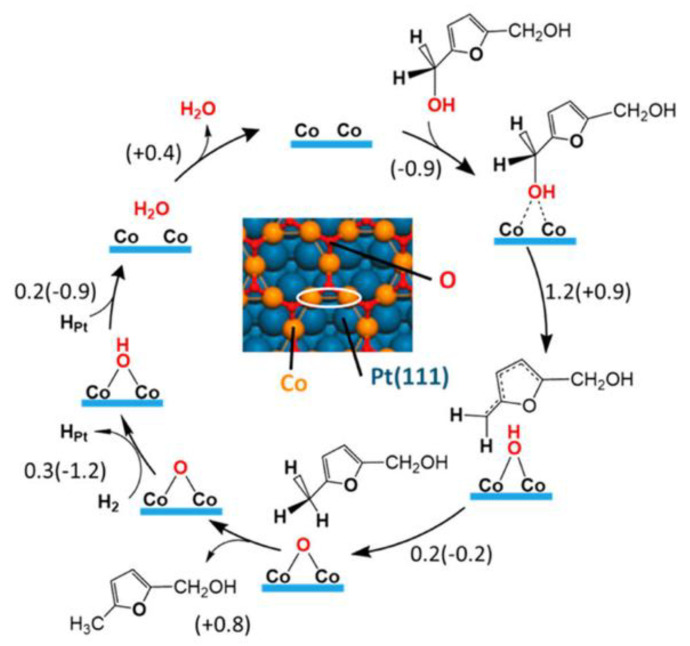
Reaction mechanism of DHMF hydrodeoxygenation to MFA on the Co_3_O_2_/Pt(111) surface based on DFT calculation. Reproduced from [87] by permission of American Chemical Society.

This synergistic phenomenon can be explained by recent studies by Luo et al. [63,87] and Chen et al. [61]. The monolayer oxide present on the surface interacted weakly with the furan ring to prevent the hydrogenatiuon of the ring and the ring opening of DMF. At the same time, it acted as an active site for the hydrogenolysis process. The proposed successive hydrogenolysis mechanism of DHMF on Pt-Co alloy is depicted in Figure 10. DHMF undergoes C–O bond cleavage on a honeycomb edge site consisting of two Co atoms, forming a loosely bound radical and an OH group. Next, an H atom transfers from the OH group to the radical, yielding MFA and a chemisorbed oxygen atom. The second hydroxymethyl group undergoes similar C–O scission, forming DMF as the final product.

### 6.4. The Role of a Support 

Catalyst support also plays an important role in the selectivity of hydrogenation reactions. The supports are usually metal oxides or carbon with the goal of maximising the specific surface area, thus giving better dispersion of the active phase. The most common support includes various types of silica, alumina and carbon. However, the use of a microporous support typically involves mass transfer issues due to diffusion limitation. In porous catalyst particles, the reacting molecules diffuse first through the fluid film surrounding the particle surface and then into the pores of the catalyst to the active sites. Similarly, the reaction products diffuse out of the catalyst grains. As an outcome of the pore diffusion in the most common reaction kinetics, the reaction rates inside the pore are lower than with the concentration level of the main bulk.

Carbon materials have been one of the most widely studied supports for catalyst preparation owing to their advantages [88]. Other than being low in cost, carbon also gives better dispersion due to a high surface area compared to other supports, such as Al_2_O_3_ and SiO_2_. Moreover, the carbon surface is relatively inert, preventing any unwanted reactions catalysed by the support surface or the reaction of the support with the active phase. Most importantly, carbon can minimise poisoning issues due to its hydrophobic nature. For example, it leads to a weaker interaction between the catalyst and the solvent. However, the chemical nature of their surface can be modified chemically to decrease the hydrophobic character by oxidising treatment [88]. For example, treatment with hydrogen peroxide and nitric acid introduces oxygen surface groups, which are responsible for the improvement of the hydrophilic character of the carbon surface. 

Priecel et al. demonstrated that CNTs as a support of Ru improve DMF yield and shorten the reaction time from 3 h to 1 h compared to carbon as a support. This owing to the superior accessibility of pores in carbon nanotubes, together with an electronic promotional effect in the carbon nanotubes, appears to be responsible for the superior activity of the catalysts supported on carbon nanotubes [67].

Ricardo et al. reported that Ru supported on materials with a high isoelectric point (basic), such as ceria, magnesia-zirconia and ɤ-alumina, results in a high yield of DHMTHF as compared to a low-isoelectric-point (acidic) support, such as SiO_2_, for selective hydrogenation of HMF [89]. This demonstrates that the basicity of the catalyst support favours the formation of ring hydrogenation of DHMF to DHMTHF. In contrast, the acidic support favours the formation of ring-opening products, such as 1,2,5-hexanetriol.

The effect of different supports on a palladium catalyst for the hydrogenation of HMF was studied by Cai et al. [90]. Acidic Pd/ɤ-Al_2_O_3_ and Pd/SiO_2_ show a similar activity as that of Pd/C, apart from the selectivity towards DHMTF, which is higher than that of Pd/C, suggesting that the acidity of the support influences the product’s selectivity. When the reaction is prolonged to 3 h, the final product is mainly DHMTHF due to the saturation of the C=C bond in DHMF (Scheme 2). As for Pd/TiO_2_, DHMF is the main product even when the reaction time is prolonged. It is speculated that by dispersing the metal on the reductive support (TiO_2_), the metal catalyst demonstrates the potential for selectively hydrogenating the carbonyl group without affecting the C=C bond 89]. In the case of Pd/HT catalyst, the basic HT support is found to restrain the dehydration reaction and inhibits the hydrogenation activity of Pd. It is also reported that HT could induce ring opening, C–O dissociation and other side reactions [91]. An extensive review on the effect of the support used and the kinetic modelling of HMF transformation into DMF has been performed by Maki et al. [92] and Esteves et al. [93].

### 6.5. The Role of Solvents

The medium of reaction also plays an important role in determining the reactivity or the product yield in a chemical reaction, especially the hydrogenation of HMF [68]. Usually, a solvent like water is preferable since it is a green solvent and no extra precaution is needed to handle it as a waste. However, not all reactions are suitable with water as the solvent. In general, a solvent can be divided into three different categories: polar protic, polar aprotic and non-polar. Polar protic solvents often display hydrogen bonding and have acidic hydrogen. These solvents have a high dielectric constant and polarity for instant water, formic acid, methanol, butanol and propanol. These types of solvents are most likely to participate in the reaction. Polar aprotic solvents lack hydrogen bonds and have moderate-to-high dielectric constants. They do not participate in the reaction, as they are relatively free in solution, making them more reactive. Common examples of such solvents are acetone, acetonitrile, THF and DMSO. Non-polar solvents are solvents such as hexane, benzene and toluene that have low dielectric constants and are not good solvents for a charged species.

A recent study by Chatterjee et al. [94] showed that a solvent with negative delta (δ) values that is capable of accepting electrons shows higher conversion of HMF. However, as the δ value increased, the conversion of HMF decreased. The authors suggested that solvent adsorption leads to the partial blocking of metal active sites. In addition, a neutral medium was preferred over a basic or an acidic medium. Water was also found to be superior to other organic solvents used for the conversion of HMF and the production of DHMF [91].

The study of the effect of solution-phase acidity on the selectivity for the hydrogenation of HMF was performed by Alamillo et al. [89]. Treatment of the HMF feed with resin led to an increase of over 20% in the selectivity of DHMTHF using Ru catalyst. The treatment with resin resulted in an increase in pH, and the increment in pH suggested that the minor impurities of acid mixed with HMF decrease the selectivity to DHMTHF. The influence of the addition of specific types of acid on the hydrogenation of HMF was also studied [89]. Levulinic acid and H_2_SO_4_ were added to the reaction mixture, and the addition of levulinic acid led to a decrease in DHMTHF yield, while H_2_SO_4_ resulted in a significant decline in DHMTHF selectivity from 76% to 9%. It was suggested that HMF and DHMF undergo acid-catalysed degradation, thus leading to low selectivity [89].

The study of the hydrogenation of HMF using different solvents was also carried out by Alamillo et al. using a water-1-butanol biphasic system, water, a mixture of THF and water and THF-alcohol [89]. It was found that HMF and DHMF are completely converted in each of the reactions. The selectivity of DHMTHF decreased when pure water was used, indicating the presence of additional degradation pathways in the presence of water. Detailed reviews on the effect of solvents were discussed by Wang et al. [95].

## 7. Conclusions

Selective hydrogenation of HMF to DMF has been extensively explored by many researchers, and some of them were able to achieve high conversion as well as a high yield of DMF under certain reaction conditions. However, the main challenge is to reduce the operational cost by using cost-effective catalysts as an alternative to the expensive noble metals. Overall, the approach of using bifunctional catalysts is an important direction for catalysis in general and particularly for reactions that require significantly different types of elementary steps as part of an overall reaction. For this reason, efforts to use rational design of interfaces will become important for supplementing the promising initial work in demonstrating bifunctional catalysis for catalytic transformations of biomass-derived 5-hydroxymethylfurfural to a potential liquid fuel, 2,5-dimethylfuran. Apart from the different types of metal, it was also observed that reaction conditions, namely the type of solvent, H_2_ donor, reaction temperature, reaction time and H_2_ pressure, play an important role in influencing the HMF conversion and DMF yield.

Future studies should focus on more variety of inexpensive metal combinations as an alloy and sustainable support without compromising the high yield of DMF. For example, the combination of non-noble metals, such as Ni, Co, Cu and Fe, would be interesting since Ni and Co are capable of converting HMF to DMF, although specific metal ratios and loading are crucial in resulting in a better catalyst. A kinetic study would be helpful to learn the adsorption behaviour of HMF and its intermediates, which would provide some insights into the principles of selectivity control that can guide catalyst selection.

## Figures and Tables

**Table 1:** Comparison of the fuel properties of DMF versus gasoline and ethanol. Adapted from [27].

Property	DMF	Ethanol	Gasoline
Gravimetric oxygen content (%)	16.7	34.8	0
Density at 20 °C (kg·m^−3^)	889.7	790.9	744.6
Water miscibility at 25 °C (gL^−1^)	2.3	Miscible	Immiscible
Boiling point (°C)	93	78	32–200
Energy density (MJ/L)	30	23.4	31
RON	119	110	95.8
Auto-ignition temperature (°C)	286	423	257

**Table 2:** Overview of DMF production from HMF in the literature.

Entry	Catalyst	Conditions					HMF Conversion (%)	DMF Yield (%)	Ref.
		Solvent	H_2_ Donor	Temperature (°C)	Time (h)	Pressure (bar)			
1	CuCrO_4_	1-Butanol	H_2_	220	10	6.8	100	61	[13]
2	CuRu/C	1-Butanol	H_2_	220	10	6.8	100	71	[13]
3	Ru/C	THF	Formic acid	75	15	-	-	32	[39]
4	Ru/K-OMS-2	1-Butanol	H_2_	220	6	10	90	33	[42]
5	Ru/C	THF	H_2_	200	2	20	100	94.7	[43]
6	Pd/C	Dioxane	Formic acid	120	15	-	100	>95	[44]
7	Ru/C	1-Butanol	H_2_	260	1.5	-	99.8	60.3	[45]
8	Pd/C	[EMIM]Cl	H_2_	120	1	62	47	15	[46]
9	Pd/C/H_2_SO_4_	THF	Formic acid	70	15	-	100	95	[47]
10	Cu-PMO	Methanol	Methanol	300	0.75	-	100	34	[48]
11	Ru/C	Isopropyl alcohol	Isopropyl alcohol	190	6	-	100	81	[49]
12	Pd/C	Water:CO_2_	H_2_	80	2	10	100	100	[50]
13	Ru/Co_3_O_4_	THF	H_2_	130	24	7	99	93.4	[51]
14	PtCo@HCS	1-Butanol	H_2_	180	2	10	100	98	[52]
15	PdAu/C	THF	H_2_	60	6	atm	100	96	[53]
16	Ni-W2C/AC	THF	H_2_	180	3	40	100	96	[54]
17	Pd/Fe_2_O_3_	2-Propanol	2-Propanol	180	-	15 ^a^	98	72	[55]
18	Cu-Ru-PMO	Ethanol	H_2_	220	6	50	100	79 ^b^	[56]
19	Pd/C/Zn	THF	H_2_	150	8	8	>99	85	[57]
20	Ru/Hydrotalcites	2-Propanol	H_2_	220	4	10	100	58	[58]
21	Ni/Co_3_O_4_	THF	H_2_	130	24	10	99	76	[59]
22	Ni-Al_2_O_3_	Dioxane	H_2_	180	4	12	100	92	[60]
23	Cu-Co@C	Ethanol	H_2_	180	8	50	100	99.4	[61]
24	Pt/rGO	1-Butanol	H_2_	120	2	30	100	73.2	[62]
25	Pt-Ni/C	1-Propanol	H_2_	200	5 ^c^	33	100	98	[63]
26	Ni-Fe/CNT	n-Butanol	H_2_	200	3	30	100	91.3 ^d^	[64]
27	Ru/NaY zeolite	THF	H_2_	220	1	15	100	78	[65]
28	CuZn	CPME	H_2_	220	6	20	100	97 ^b^	[66]
29	Ru/CNT	Dioxane	H_2_	150	1	20	100	83.5	[67]

^a^ N_2_ pressure was used. ^b^ DMF + DMTHF. ^c^ Continuous-flow reactor. ^d^ Selectivity.

## Data Availability

Not applicable.
